# Cadmium Toxicity Effects on Histone Modifiers, Enzyme Activity and Adipokines in Human Adipose Tissue Cells

**DOI:** 10.3390/molecules31061056

**Published:** 2026-03-23

**Authors:** Victor Tadeu Gonçalves Plata, Júlia Fernandes Barcella, Raphael Justa Saran, Artur Francisco da Silva Neto, Yasmin Alaby Martins Ferreira, Andressa Bolsoni-Lopes, Lila Missae Oyama, Lucia Maria Armelin-Correa, Maria Isabel Cardoso Alonso-Vale

**Affiliations:** 1 Post-Graduate Program in Chemical Biology, Institute of Environmental Sciences, Chemical and Pharmaceutical, Federal University of São Paulo—UNIFESP, Diadema 09913-030, Brazil; victor.plata@unifesp.br (V.T.G.P.); yasmin.alaby@unifesp.br (Y.A.M.F.); lucia.correa@unifesp.br (L.M.A.-C.); 2 Department of Biological Sciences, Institute of Environmental Sciences, Chemical and Pharmaceutical, Federal University of São Paulo—UNIFESP, Diadema 09972-270, Brazil; julia.barcella@unifesp.br (J.F.B.); raphael.justa@unifesp.br (R.J.S.); 3 Post-Graduate Program in Nutrition, Paulista School of Medicine, Federal University of São Paulo—UNIFESP, São Paulo 04023-062, Brazil; artur.francisco@unifesp.br (A.F.d.S.N.); lmoyama@unifesp.br (L.M.O.); 4 Graduate Program in Physiological Sciences, Health Sciences Center, Federal University of Espírito Santo, Vitória 29043-900, Brazil; andressa.lopes@ufes.br

**Keywords:** adipose tissue, mesenchymal stem cells, heavy metals, inflammation, adipogenesis

## Abstract

Environmental exposure to heavy metals, particularly cadmium (Cd), has been increasingly associated with obesity, metabolic dysfunction, chronic inflammation, and related disorders such as type 2 diabetes and cardiovascular diseases. Adipose tissue (AT), a paracrine and endocrine organ central to systemic energy and inflammatory homeostasis, is a major site of heavy metal accumulation and a key target of Cd toxicity. However, the mechanisms by which Cd disrupts adipocyte function, especially through epigenetic pathways, remain poorly understood. In this study, we investigated the effects of Cd on epigenetic regulators, antioxidant enzyme activity, inflammatory mediators, and adipogenic programming in human adipose-derived stromal/stem cells (hASCs) and differentiated adipocytes. Cd exposure altered histone modifiers associated with lysine 27 of histone 3 (H3K27), disrupted redox balance in a concentration-dependent manner, impaired adipogenic differentiation and lipid accumulation, and modulated inflammatory and adipokine responses according to differentiation stage and Cd concentration. Our findings suggest that Cd compromises adipose cell homeostasis through mechanisms involving epigenetic dysregulation, oxidative stress imbalance, and altered adipogenic and inflammatory signalling. These observations point to possible long-term metabolic consequences of environmental Cd exposure due to its accumulation in adipose tissue.

## 1. Introduction

The impact of heavy metals in the environment has been a global concern for the past few decades. Metals such as lead, mercury, and cadmium (Cd), even at small concentrations, can cause dangerous effects for human health and the environment [1]. The main anthropogenic sources of Cd in the environment are derived from non-ferrous metal mining and refining, disposal and incineration of waste, manufacturing of phosphate fertilizers and their application, and the combustion of fossil fuels [2]. Smoking habits greatly increase exposure to Cd, since tobacco leaves have naturally high amounts of Cd. It has been estimated that tobacco smokers are exposed to 1.7 μg of Cd per cigarette, and about 10% is inhaled when smoked [2]. Cd is classified as a group I carcinogen by the International Agency for Research on Cancer (IARC) [3].

Cd possesses a very slow elimination rate (20–40 years), leading to significant accumulation in multiple tissues, including adipose tissue (AT) [4,5]. Once accumulated, Cd can exert its toxic effects partly by mimicking essential metals such as zinc and calcium. By displacing these ions from key molecules—such as zinc-finger proteins—Cd may disrupt cellular pathways and disturb homeostasis [6,7]. Indeed, Cd accumulation in AT has been shown to affect the PPAR pathway, increase lipolysis, and alter serum lipid profiles in mice, with human studies corroborating that higher blood Cd correlates with increased cholesterol and triglycerides [8]. Cd also impairs adipogenesis by downregulating key regulators such as C/EBP and PPARG [5], while promoting oxidative stress, inflammation, and insulin resistance [8,9].

In line with these metabolic disturbances, Cd exposure has been shown to induce macrophage recruitment to white adipose tissue (WAT) and upregulate the pro-inflammatory chemokine CCL2/MCP-1 in mice [10], which plays a prominent role in obesity-related risks [11]. In human WAT, Gasser et al. (2022) demonstrated that Cd induces pro-inflammatory gene expression, disrupts adipocyte function, and impairs the homeostasis of small molecules, vesicle trafficking, and enzyme activity [12]. Furthermore, Cd adversely affects solute carrier (SLC) transporters and metallothioneins involved in trace element ion trafficking, contributing to metabolic disorders [13]. These functional alterations are consistent with transcriptomic studies showing widespread gene expression changes in the WAT of Cd-exposed mice [5].

WAT is a highly complex tissue composed of lipid-filled cells called adipocytes and the stromal vascular fraction (SVF), which includes endothelial cells, fibroblasts, immune cells, preadipocytes, and mesenchymal adipose stem cells (mASCs). These cells contribute to the synthesis of extracellular matrix components that together establish specialized microenvironments within distinct anatomical adipose depots [14]. AT expands through two main mechanisms: hypertrophy, which involves an increase in the size of existing adipocytes, and hyperplasia, the formation of new adipocytes via differentiation of mASCs in adipocytes [11].

During adipogenesis, epigenetics plays a crucial role in regulating cell differentiation and gene expression. In this context, Cd has been observed to alter the expression of histone modifiers, such as H3K4 and H3K9 demethylases [12,15]. It was demonstrated that EZH2-induced H3K27me3—mediated by the enhancer of polycomb repressive complex 2 subunit (EZH2), a histone methyltransferase—at Wnt gene promoters facilitated adipogenic differentiation of murine preadipocytes [16]. Moreover, the human JmjC-domain-containing proteins KDM6A and KDM6B (also known as UTX and JMJD3) demethylate H3K27me3 marks. Ectopic expression of KDM6B has been shown to strongly decrease H3K27me3 levels and cause delocalization of polycomb proteins in vivo [17]. In their review, Zhao et al. (2024) highlight that depletion of KDM6B significantly increases adipogenic differentiation [18]. Additionally, nickel upregulates KDM6B expression in kidney cancer cells [17]. Since Cd can occupy similar binding sites as nickel in proteins—particularly at zinc-binding sites, as both are transition metals that can substitute for zinc—Cd exposure may also affect KDM6B expression. Furthermore, H3K27 acetylases such as EP300 and CREBBP play an important role in activating key transcription factors in adipose tissue [19] and have been shown to be affected by exposure to heavy metals such as lead and Cd [20].

A key driver of epigenetic reprogramming is the accumulation of reactive oxygen species (ROS) [21,22]. Cd is a well-established inducer of oxidative stress, primarily by depleting cellular antioxidant defenses (e.g., glutathione) and promoting ROS generation [9,23]. This redox imbalance can, in turn, modulate epigenetic regulators via ROS-sensitive pathways. For instance, ROS can induce the expression of the histone demethylase KDM6B [21,24], which removes repressive H3K27me3 marks and facilitates activating H3K27ac marks, thereby linking oxidative stress to epigenetic changes, promoting the transcription of target genes involved in inflammation and antioxidant responses.

Based on these observations, we hypothesized that exposure to non-cytotoxic concentrations of Cd modulates the expression of H3K27 histone modifiers—particularly KDM6B via ROS-dependent mechanisms—with consequent effects on adipogenic differentiation and adipokine secretion in human adipose-derived stem cells (hASCs) and differentiated adipocytes. To test this hypothesis, we examined: (i) the expression of H3K27 histone modifiers and global H3K27me3/H3K27ac marks; (ii) the activity of antioxidant enzymes and protein carbonyl content as a marker of oxidative damage; (iii) the expression and secretion of adipokines and inflammatory mediators; and (iv) the impact on adipogenic differentiation, including the expression of key adipogenic genes and lipid accumulation. By integrating these complementary endpoints in both undifferentiated and differentiated cellular contexts, this study aims to provide mechanistic insights into the potential link between Cd exposure and AT dysfunction.

## 2. Results

### 2.1. hASCs Viability After Exposure to Different Cd Concentrations

**Figure 1:**
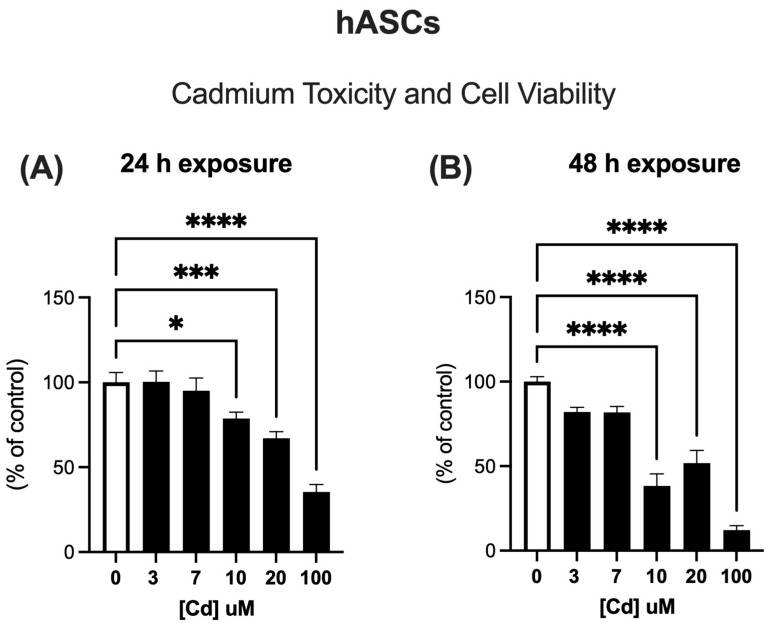
Cell viability assay on cells exposed to Cd using MTT. hASCs were cultivated until high confluence and then treated with Cd at increasing concentrations (0, 3, 7, 10, 20 and 100 µM) for 24 (**A**) and 48 (**B**) hours. Cell viability was evaluated by MTT assay, as described in Section 4 Data represent mean ± SEM from n = 4 independent human donors (biological replicates), each assayed in technical triplicate. * *p* < 0.05; *** *p* < 0.0001; **** *p* < 0.00001 (one-way ANOVA, followed by Tukey’s test).

To determine the appropriate Cd concentration in our model, hASCs were exposed to different Cd concentrations (3, 7, 10, 20, and 100 µM) for 24 (Figure 1A) and 48 h (Figure 1B) and assessed for cell viability using the MTT assay. Since the results showed that 10 µM affected cell viability, we selected two lower concentrations for subsequent experiments: a “low” concentration of 1.5 µM and a “high” concentration of 5 µM.

### 2.2. hASCs Treated with Low (1.5 µM) and High (5 µM) Cd Concentrations: Effects on Expression of H3K27 Modifiers and Epigenetic Marks

**Figure 2:**
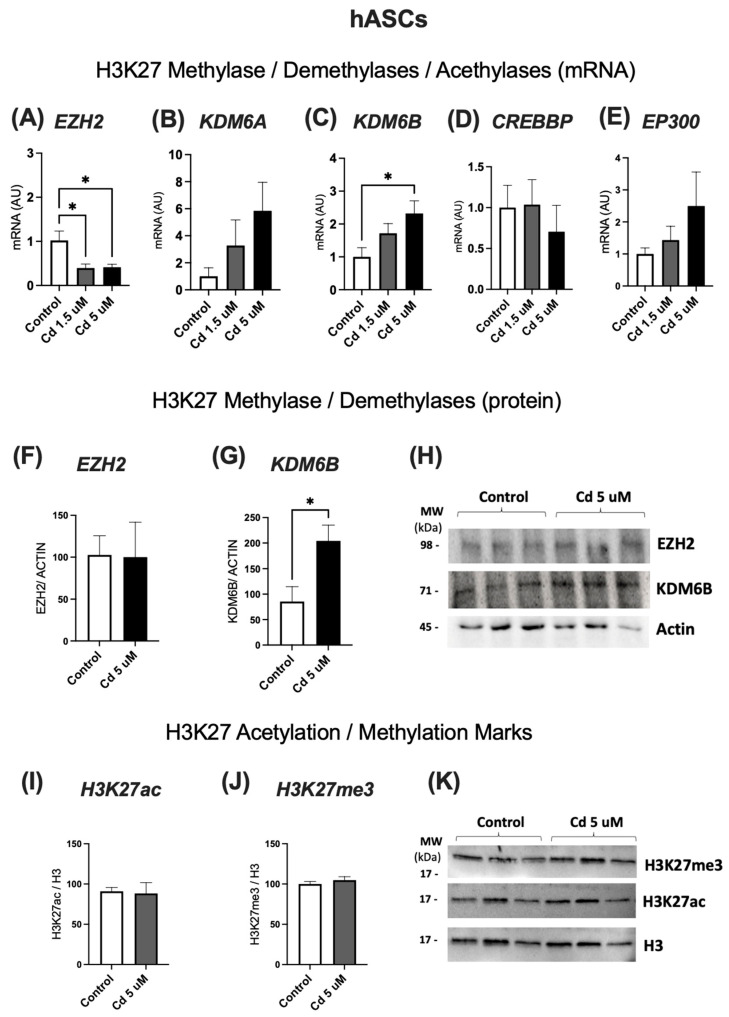
mRNA levels of epigenetic modifiers: *EZH2* (**A**), *KDM6A* (**B**), *KDM6B* (**C**), *CREBBP* (**D**) and *EP300* (**E**). hASCs were cultivated until high confluence and treated with Cd at low (1.5 µM) or high (5 µM) concentration for 10 days. Western Blot analysis of H3K27 modifiers: *EZH2* (**F**), and *KDM6B* (**G**). Graphical representation of protein expression of EZH2, KDM6B and β-actin (**H**). Western Blot analysis of H3K27 acetylation (**I**) and methylation (**J**) marks. Graphical representation of protein content of H3K27me3 and H3K27ac marks, along with total H3 protein content (**K**). GAPDH was used as the housekeeping gene. Data represent mean ± SEM from n = 4 independent human donors (biological replicates), each assayed in technical triplicate. * *p* < 0.05 (one-way ANOVA, followed by Tukey’s post-test or Student’s *t* test).

After selecting the optimal Cd concentrations, we decided to start our experiments by observing the impact of both concentrations on the H3K27 modifiers. The expression of the methylase *EZH2* was reduced on hASCs exposed to both Cd concentrations (Figure 2A), while the demethylase *KDM6B* had a significant increase on the cells treated with 5 µM, showing that Cd is causing alterations in the epigenetic mechanisms of hASCs (Figure 2C).

Next, we decided to test if the alterations in the gene expression of these enzymes resulted in an alteration in the expression of the respective proteins. While we did not observe a change in the protein levels of EZH2, KDM6B showed an increase in the levels of protein expression, just like it was seen in our previous experiment regarding gene expression (Figure 2G).

Considering that our results showed that Cd altered the expression of some histone modifiers, we also evaluated the H3K27 acetylation and methylation marks. However, there was no difference in both epigenetic marks between the group treated with Cd and the control group, suggesting that the changes in *EZH2* and *KDM6B* expression may have affected only specific regions on the genome, and did not change the global acetylation and methylation marks (Figure 2K).

### 2.3. hASCs Treated with Low (1.5 µM) and High (5 µM) Cd Concentrations: Effects on Enzymatic Antioxidant System and Expression of Pro-Inflammatory Mediators

**Figure 3:**
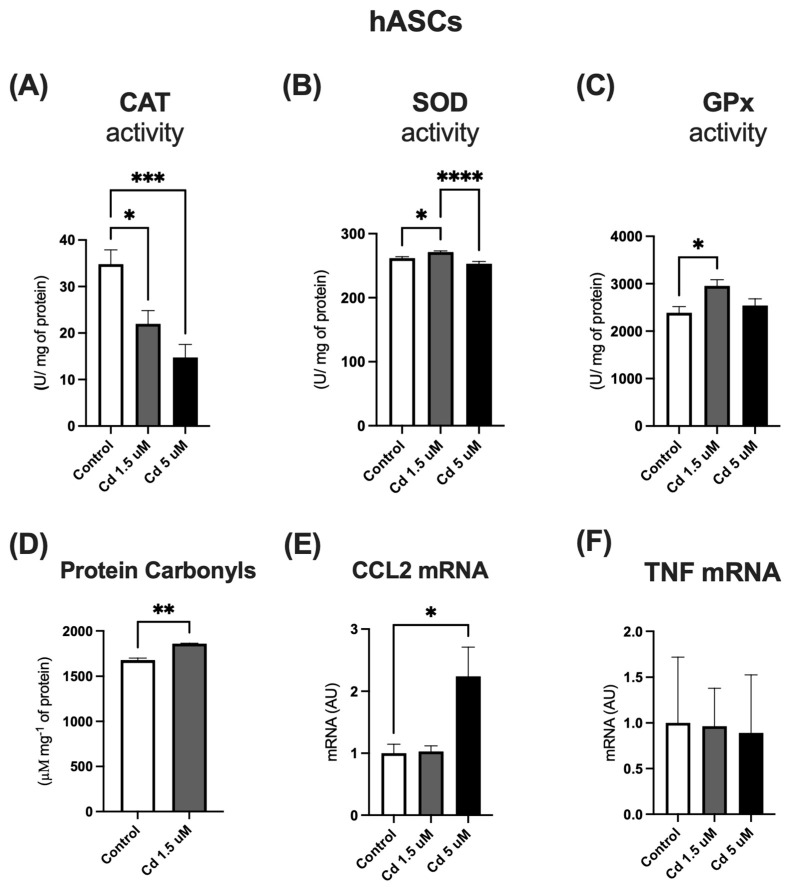
Enzymatic activity of Catalase (**A**), Superoxide Dismutase (SOD) (**B**) and Glutathione Peroxidase (GPx) (**C**). Protein carbonyl content (**D**). mRNA levels of pro-inflammatory mediators *CCL2* (**E**) and *TNFa* (**F**). All experiments were performed using hASCs treated with low (1.5 µM) or high (5 µM) Cd concentrations for 10 days. GAPDH was used as the housekeeping gene. Data represent mean ± SEM from n = 4 independent human donors (biological replicates), each assayed in technical triplicate. * *p* < 0.05; ** *p* < 0.001; *** *p* < 0.0001; **** *p* < 0.00001 (one-way ANOVA, followed by Tukey’s post-test).

Our results showed a significant decrease in catalase (CAT) activity in Cd-treated groups compared to the control (Figure 3A). Superoxide dismutase (SOD) and glutathione peroxidase (GPx) activities were also significantly altered, exhibiting a biphasic response: both enzymes showed a significant increase at the lower Cd concentration (1.5 µM), but this effect was no longer observed at the higher concentration (5 µM) (Figure 3B,C). These findings suggest that Cd interferes with the enzymatic antioxidant system in a concentration-dependent manner. Notably, protein carbonyl content—a direct marker of oxidative damage—was already elevated at the lowest Cd concentration tested (1.5 µM), as shown in Figure 3D.

Furthermore, we assessed the impact of prolonged Cd exposure on the inflammatory profile of hASCs. Following a 10-day treatment, Cd exposure specifically upregulated the expression of the pro-inflammatory chemokine CCL2 (Figure 3E). In contrast, TNF-α gene expression remained unchanged (Figure 3F). This selective inflammatory response was observed under conditions that did not compromise cell viability, as confirmed by MTT assays.

### 2.4. hASCs Treated with Low (1.5 µM) Cd Concentration During Adipogenic Differentiation: Effects on the Expression of Adipogenic Markers

To evaluate the impact of prolonged, low-level Cd exposure on adipose tissue development, we induced adipogenic differentiation of hASCs in the continuous presence of Cd (1.5 µM). To model a potential synergistic effect with inflammation—a common comorbidity in metabolic disorders—we co-treated some groups with lipopolysaccharide (LPS), which served as a positive control for inflammatory response.

**Figure 4:**
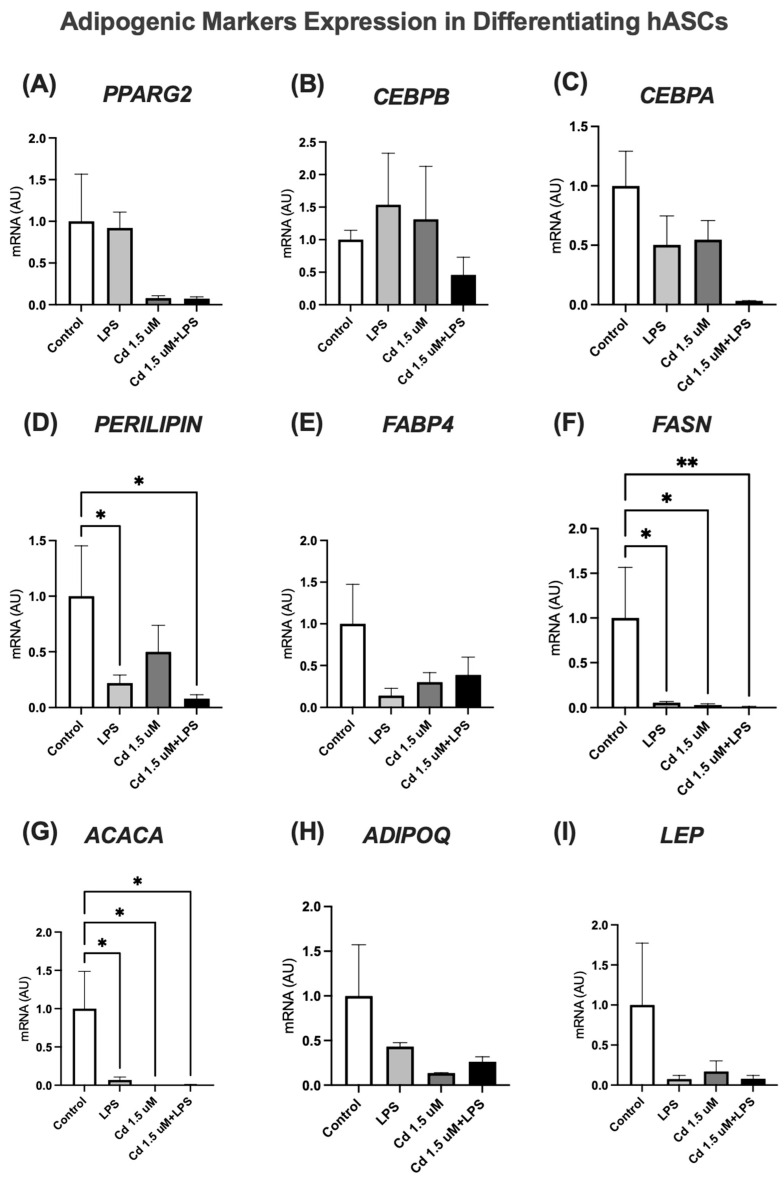
Gene expression of adipogenic markers in differentiating hASCs, treated with Cd and/or LPS for 7 days. *PPARG2* (**A**), *CEBPB* (**B**), *CEBPA* (**C**), *PERILIPIN/PLIN1* (**D**), *FABP4* (**E**), *FASN* (**F**), *ACACA* (**G**), *ADIPOQ* (**H**) and *LEP* (**I**). The cells were cultured until they reached confluence (D-2). After 2 days (D0), the cells were differentiated with an adipogenic cocktail, in the presence or absence of Cd (1.5 µM) and/or lipopolysaccharide (LPS, 1 μg/mL) and maintained until day 7 post-differentiation. GAPDH was used as the housekeeping gene. Data represent mean ± SEM from n = 4 independent human donors (biological replicates), each assayed in technical triplicate. * *p* < 0.05; ** *p* < 0.001 (one-way ANOVA, followed by Tukey’s post-test).

Analysis of the adipogenic transcriptional cascade revealed a profound disruption (Figure 4). While the expression of the early factor CEBPB was not significantly altered (Figure 4B), the master regulators of terminal differentiation, PPARγ and CEBPA, showed a downregulation in response to Cd and/or LPS exposure (Figure 4C,E). This attenuation of the core adipogenic program was further reflected in the expression of key functional markers.

The expression of PLIN1 (perilipin-1), a gene essential for lipid droplet formation and stabilization, was significantly reduced (Figure 4D). Similarly, genes encoding rate-limiting enzymes for de novo lipogenesis, ACACA and FASN, were also downregulated in treated cells (Figure 4F,G). This coordinated suppression indicates a severe compromise in both lipid storage capacity and overall adipocyte maturation.

Critically, the expression of late adipogenic markers, which define the functional adipocyte phenotype, was also impaired. We observed a significant decrease in the expression of the insulin-sensitizing hormone adiponectin (ADIPOQ) and the satiety-regulating hormone leptin (LEP) (Figure 4H,I).

### 2.5. Oil Red O Staining on Subcutaneous hASCs During Differentiation

The assessment of lipid accumulation after staining with Oil Red O was performed to better estimate the adipogenic potential of preadipocytes induced to differentiate in the presence of Cd, with or without LPS.

**Figure 5:**
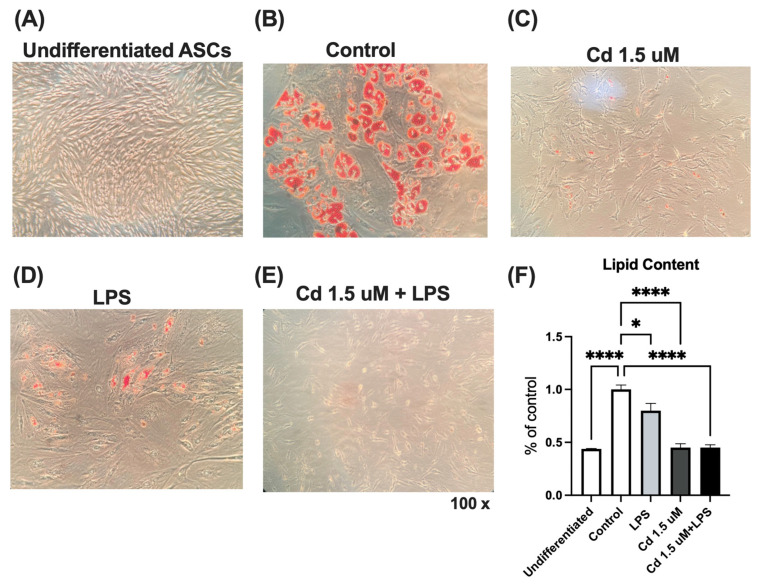
Effect of Cd on intracellular lipid accumulation in differentiated hASCs. hASCs were cultured until 100% confluency and induced to differentiate by adding an adipogenic cocktail for 7 days, in the presence (or absence) of LPS (1 µg/mL) and/or Cd (1.5 µM). All treatments were added from day 0 of differentiation. Cells were fixed with formalin and then stained with Oil Red O, as described in the methods. (**A**–**E**) Representative images of stained cells after 7 days with their respective treatments, visualized using an inverted microscope. (**F**) Intracellular stained lipid droplets were eluted with isopropanol and quantified by spectrophotometric analysis at 540 nm. Lipid content is expressed as a percentage of the control (untreated) group. Data represent mean ± SEM from n = 4 independent human donors (biological replicates), each assayed in technical triplicate. * *p* < 0.05; **** *p* < 0.00001 (one-way ANOVA, followed by Tukey’s post-test).

Seven days after differentiation induction, the cells were stained with oil red O and photographed. As can be seen in Figure 5, the addition of Cd to the cultures decreased the number of d2ifferentiated cells compared to the control (cells differentiated with the adipogenic cocktail, without the presence of Cd and/or LPS), evidenced by the lower amount of lipids inside them, a ~50% decrease (*p* < 0.0001) in lipid content in cells treated with 1.5 µM of Cd (Figure 5F).

### 2.6. Differentiated Adipocytes from hASCs Treated with High (5 µM) Cd Concentration: Effects on the Expression of H3K27 Modifiers and Pro-Inflammatory Mediators

We next assessed whether chronic exposure to a higher, potentially stress-inducing Cd concentration (5 µM) would alter the inflammatory and endocrine profile of mature adipocytes. Given its established role in obesity-associated inflammation, the chemokine CCL2/MCP-1 was a primary focus. LPS was used as a positive control for inflammatory activation.

**Figure 6:**
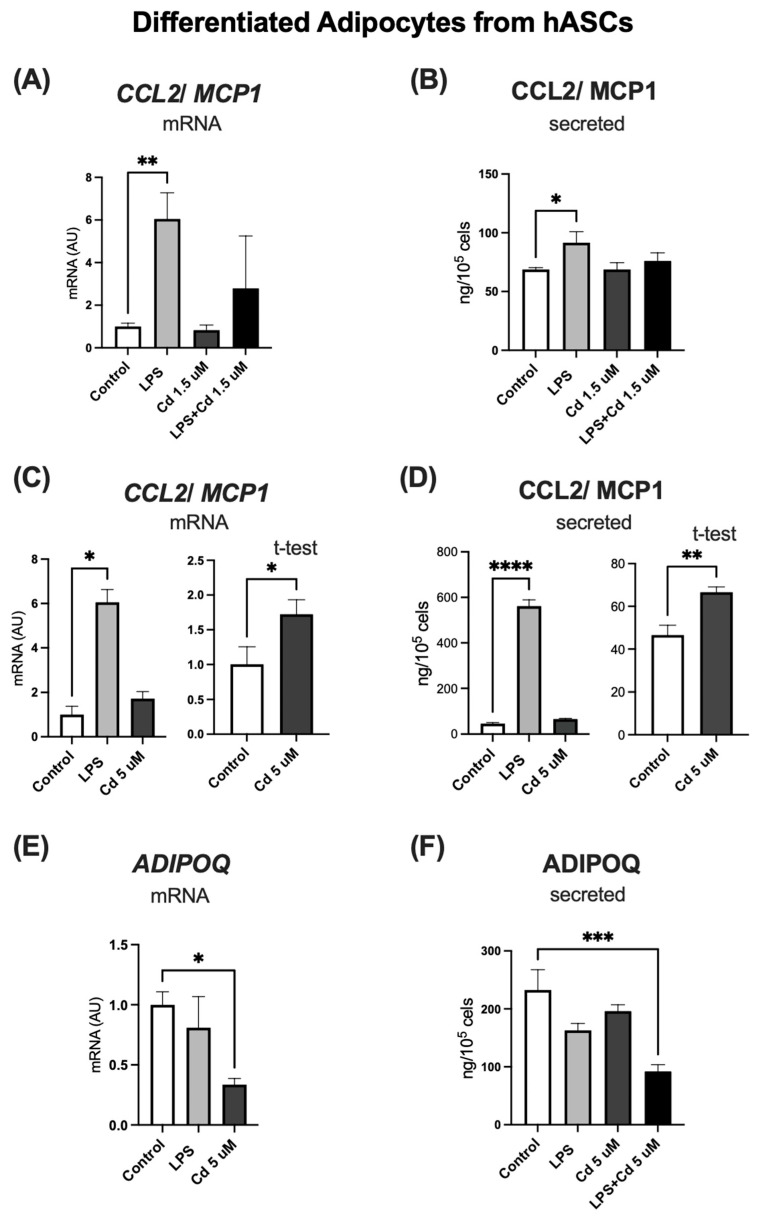
Expression and secretion of adipo(cyto)kines by hASC-derived adipocytes following chronic Cd exposure. *CCL2/MCP1* gene expression (**A**) and protein secretion (**B**) in mature adipocytes treated with low (1.5 µM) Cd concentration and/or lipopolysaccharide (LPS, 1 μg/mL). *CCL2/MCP1* gene expression (**C**) and protein secretion (**D**) in mature adipocytes treated with high (5 µM) Cd concentration or LPS. *ADIPOQ* gene expression (**E**) and protein secretion (**F**) in mature adipocytes treated with high (5 µM) Cd concentrations and/or LPS. Primary hASCs were differentiated into adipocytes prior to treatment. Treatments were initiated on day 10 post-differentiation and maintained for 10 days. GAPDH was used as the housekeeping gene. Data represent mean ± SEM from n = 4 independent human donors (biological replicates), each assayed in technical triplicate. * *p* < 0.05; ** *p* < 0.001; *** *p* < 0.0001; **** *p* < 0.00001 (one-way ANOVA, followed by Tukey’s post-test or Student’s *t* test).

In hASCs fully differentiated into adipocytes treated with a low Cd concentration (1.5 µM) during differentiation, we observed that Cd alone did not stimulate CCL2 expression or secretion. However, when co-administered with LPS, it significantly attenuated the LPS-induced secretion of CCL2 (Figure 6A,B), suggesting a modulatory interaction at low exposure levels.

In stark contrast, direct treatment of mature adipocytes with a high Cd concentration (5 µM) triggered a robust pro-inflammatory response, marked by a significant increase in both CCL2 gene expression and protein secretion (Figure 6C,D). This high-dose exposure also significantly suppressed the expression and secretion of ADIPOQ, a key insulin-sensitizing adipokine (Figure 6E,F).

### 2.7. Histone Modifiers and Antioxidant Enzymes in Differentiated hASCs

Having established that Cd modulates the expression of specific H3K27 histone modifiers in undifferentiated hASCs, we sought to investigate whether this epigenetic dysregulation persists or is altered in mature adipocytes. To this end, we analyzed the expression of key histone-modifying enzymes in hASCs fully differentiated into adipocytes following chronic Cd exposure (5 µM).

**Figure 7:**
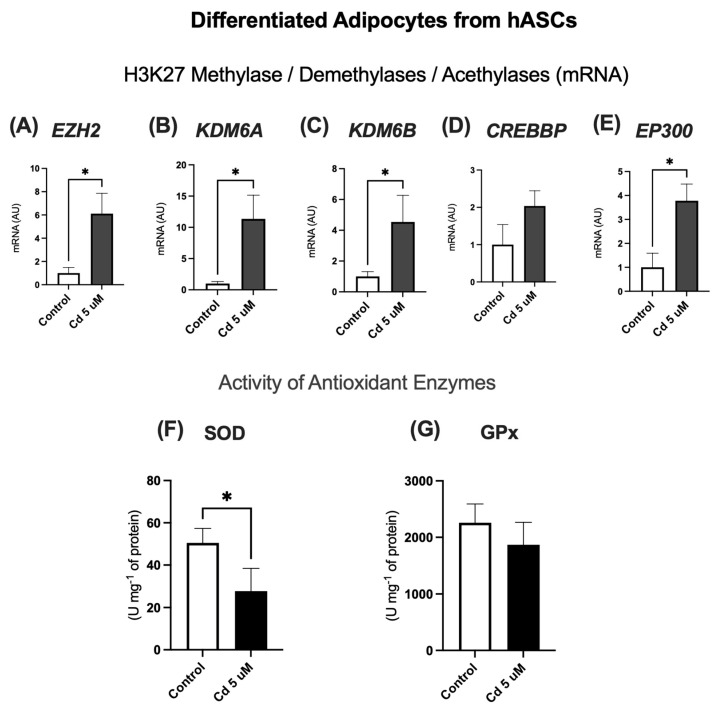
Expression of genes encoding histone-modifying enzymes and activity of antioxidant enzymes in hASC-derived adipocytes following chronic Cd exposure. Gene expression of the methyltransferase EZH2 (**A**), the demethylases KDM6A (**B**) and KDM6B (**C**), and the acetyltransferases CREBBP (**D**) and EP300 (**E**). Enzymatic activity of Superoxide Dismutase (SOD) (**F**) and Glutathione Peroxidase (GPx) (**G**). Primary hASCs were differentiated into adipocytes prior to treatment. Adipocytes were treated with 5 µM Cd, starting on day 10 post-differentiation, and the treatment was maintained for 10 days. Gene expression data are normalized to GAPDH. Data represent mean ± SEM from n = 4 independent human donors (biological replicates), each assayed in technical triplicate. * *p* < 0.05 (Student’s *t* test).

We observed a distinct transcriptional response in the differentiated state. The gene expression of most histone modifiers investigated—namely EZH2, KDM6A, KDM6B, and EP300—was significantly upregulated in Cd-treated adipocytes (Figure 7A–C,E). In contrast, CREBBP expression remained unchanged (Figure 7D).

We next extended this comparative analysis to the activity of antioxidant enzymes. Similar to the pattern observed in undifferentiated hASCs, Superoxide Dismutase (SOD) activity was significantly reduced in Cd-exposed adipocytes (Figure 7F). The activity of Glutathione Peroxidase (GPx) also showed a decreasing trend, although it did not reach statistical significance (Figure 7G).

## 3. Discussion

The present study suggests that Cd, at non-cytotoxic concentrations, may affect adipose cell homeostasis. This may be coordinated effects on epigenetic regulation, redox balance, adipogenic programming, and inflammatory signalling in hASCs and differentiated adipocytes. By analysing the distinct stages of adipocyte differentiation, our findings indicate that AT is not only a passive site of Cd accumulation, but a critical target of its long-term metabolic toxicity.

One of the most striking observations of this study is the differential modulation of H3K27-associated histone modifiers by Cd exposure. In undifferentiated hASCs, Cd reduced *EZH2* expression while inducing *KDM6B*, particularly at the higher concentration (5 µM), with consistent changes at the protein level for KDM6B. EZH2-mediated H3K27 trimethylation is classically associated with transcriptional repression of differentiation-associated genes, whereas KDM6B promotes transcriptional activation through H3K27me3 removal [25]. KDM6B acts as a key target of LPS-induced inflammation (via NF-κB) in hASCs, being significantly elevated, which contributes to unfavourable epigenetic changes [26]. The reciprocal regulation of these enzymes suggests that Cd perturbs the epigenetic balance governing lineage commitment in hASCs. Importantly, despite these alterations, global H3K27me3 and H3K27ac levels remained unchanged, indicating that Cd may induces locus-specific chromatin remodelling rather than widespread epigenomic reprogramming. This interpretation aligns with previous reports showing that heavy metals can selectively alter histone modifier expression without necessarily changing global marks [17]. However, it is important to acknowledge that our targeted approach—focusing on a limited set of epigenetic regulators—precludes identification of the specific genomic loci affected by Cd. Without genome-wide techniques such as ChIP-pPCR or CUT&RUN, the full epigenetic landscape remains uncharacterized, and our conclusions regarding locus-specific effects remain hypothetical. Future studies employing unbiased genome-wide approaches are essential to precisely map Cd-induced epigenetic changes at key adipogenic and inflammatory gene loci.

The effects of Cd-induced epigenetic alterations became even more pronounced in differentiated adipocytes, which showed increased expression of most H3K27 modifiers, including EZH2, KDM6A, KDM6B, and EP300. This marked divergence from—and exacerbation relative to—the undifferentiated state underscores that the epigenetic response to Cd is highly contextual and dependent on the differentiation status of adipose cells. Given that mature adipocytes and progenitor cells fulfil distinct roles in AT metabolic regulation, such stage-specific epigenetic dysregulation may contribute to long-term alterations in adipose tissue plasticity and function following environmental Cd exposure [12].

Our finding of a marked upregulation of the histone demethylase KDM6B in Cd-treated hASCs led us to hypothesize that Cd disrupts cellular homeostasis through epigenetic mechanisms involving this key H3K27 modifier. KDM6B expression is likely driven by ROS-sensitive pathways, such as STAT6, as reactive oxygen species are established mediators of epigenetic reprogramming [21,22]. Functionally, KDM6B catalyzes the removal of repressive histone marks (H3K27me3) and facilitates the addition of activating ones (H3K27ac), thereby promoting gene transcription. In our model, this epigenetic shift promoted a pro-inflammatory response, characterized by increased gene expression and secretion of CCL2/MCP-1, which aligns with established links between KDM6B activity and inflammatory pathways [26,27].

Crucially, this KDM6B-mediated reprogramming occurred within a context of Cd-induced redox imbalance, characterized by a failure to neutralize reactive oxygen species (ROS) by antioxidant enzymes [24]. To assess the impact of Cd on the enzymatic antioxidant system—a topic still underexplored in hASCs—we evaluated key enzymes activities. Cd exposure interfered with this defence. We observed a marked and consistent reduction in CAT activity. The response of SOD and GPx was biphasic; an initial increase at a lower Cd concentration may reflect a compensatory adaptation, while its loss at a higher concentration suggests enzyme inhibition or exhaustion of antioxidant capacity [28]. Given that Cd promotes ROS generation indirectly by impairing mitochondrial function and antioxidant enzymes [28], the attenuation of CAT is particularly significant for hydrogen peroxide accumulation.

This evidence supports a self-propagating cycle: Cd-induced ROS can drive KDM6B expression, leading to pro-inflammatory gene activation. Concurrently, the suppression of key antioxidant defences (like CAT) and the insufficiency of partial compensatory responses (SOD, GPx) exacerbate redox imbalance further fueling the epigenetic-inflammatory cascade [24]. Thus, by modulating KDM6B expression within a compromised antioxidant environment, Cd initiates a pathway that concurrently amplifies inflammatory signalling and undermines cellular resilience, leading to long-term dysfunction. Interestingly, one study showed that silencing KDM6B attenuated the increase in ROS levels in LPS and IFN−γ−treated Raw264.7 cells [29].

The increase in protein carbonyl content observed in Cd-treated hASCs suggests oxidative damage to cellular proteins. Concurrently, the elevation of antioxidant enzyme activities, such as SOD and GPx, likely reflects a compensatory cellular response to increased oxidative stress [30]. Moreover, future studies employing antioxidant interventions (e.g., N-acetylcysteine) or genetic approaches (e.g., KDM6B knockdown or overexpression) are necessary to definitively establish whether ROS drive KDM6B expression in response to Cd exposure.

Beyond the classical enzymatic antioxidant defenses (CAT, SOD, and GPx), thiol-based redox systems play a central role in maintaining cellular redox homeostasis [31,32,33]. Key among these are the thioredoxin (Trx) and glutaredoxin (Grx) systems, which regulate protein redox status through reversible thiol-disulfide exchange reactions. By reducing oxidized cysteine residues, they are vital for antioxidant defense, redox signalling, and cell survival, as demonstrated in models like SH-SY5Y neuroblastoma cells [34]. Notably, Cd is known to strongly interfere with these systems in various cell types; for instance, it inhibits thioredoxin reductase (TrxR) and disrupts Grx-dependent pathways due to its high affinity for thiol groups, as shown in Jurkat T-lymphocytes [35]. Such impairment compromises the cell’s capacity to maintain proteins in their reduced, functional state, thereby exacerbating oxidative stress. However, whether Cd similarly dysregulates these critical thiol-based systems in AT or its precursor cells remains an important question to be explored.

Cd exposure during adipogenic differentiation resulted in a clear impairment of adipogenic programming. Specifically, the overall adipogenic cascade was negatively affected, as evidenced by reduced gene expression of key adipocyte markers, including *PLIN1*, *ACACA*, *FASN*, *ADIPOQ*, and *LEP*. These transcriptional changes were functionally reflected in a pronounced reduction in lipid accumulation, indicating defective adipocyte maturation. Importantly, the suppression of lipogenic genes suggests that Cd interferes not only with adipocyte differentiation but also with the metabolic capacity of mature adipocytes to synthesize and store lipids [5,36]. Consequently, this dysfunctional phenotype—characterized by impaired lipid storage and altered adipokine secretion—resembles features of unhealthy adipose tissue expansion, which is commonly associated with ectopic lipid deposition, insulin resistance, and metabolic disease [37]. Our findings thus demonstrate that low-dose Cd exposure, particularly under inflammatory conditions, disrupts adipogenesis at multiple stages: it impairs the terminal transcriptional program, suppresses genes critical for lipid droplet biology and synthesis, and ultimately hinders the acquisition of the mature adipocyte endocrine phenotype.

The influence of Cd on *CCL2/MCP-1* expression is also noteworthy. This monocyte chemoattractant is a widely recognised marker of inflammation in AT, being related to macrophage infiltration and the development of insulin resistance [11]. The observation that Cd modulated *CCL2* expression differently depending on the stage of cell differentiation and the presence of LPS suggests a modulatory effect of the metal, which deserves further investigation. Our findings reinforce the idea that chronic exposure to Cd, even at low concentrations, poses a significant risk to human adipose tissue homeostasis. Considering that Cd accumulates in tissues with slow cell renewal, such as adipose tissue, and that its half-life can exceed decades, the epigenetic and functional effects observed here may have lasting implications for individuals exposed occupationally or via lifestyle (e.g., smoking, consumption of contaminated food) [2,5].

By demonstrating that Cd affects both progenitor cells and mature adipocytes through distinct but overlapping mechanisms, this study provides new insight into how environmental Cd exposure may contribute to adipose tissue dysfunction and metabolic disease. Unlike previous studies that focused on effects of epigenetic mechanisms, such as histone modifications on adipogenesis [18], and the influence of Cd on adipogenesis [10], and dysfunction of adipose tissue [38,39], our study provides the first evidence of Cd effects on the expression of the epigenetic modifiers (specifically *KDM6B*, *KDM6A*, and *EZH2*) in primary human adipose-derived stem cells (hASCs) and differentiated human adipocytes. Furthermore, we integrate for the first time the triad of epigenetic regulation, redox balance, and adipokine secretion in a human adipose tissue cellular model, offering a more physiologically relevant perspective on Cd-induced metabolic disruption.

Although concentrations of 1.5 µM and 5 µM may appear relatively low, they fall within the range commonly employed in mechanistic in vitro studies [12]. Such concentrations are particularly relevant for investigating dose–response relationships and for identifying molecular pathways affected by Cd. Moreover, chronic exposure to Cd in adipose tissue, even at low concentrations, has been reported to dysregulate the secretion of adipokines such as leptin and adiponectin, highlighting the toxic effects of Cd on adipose tissue function [40]. It is worth mentioning that extrapolation to human environmental exposure requires caution and that future studies should explore lower, more environmentally relevant concentrations in chronic exposure models.

Some limitations of this study should be acknowledged. Our targeted approach reveals changes in key epigenetic modifiers, but the analysis focused on a selected set of epigenetic regulators and antioxidant enzymes. Genome-wide approaches would be necessary to identify specific loci affected by Cd-induced chromatin remodelling and to fully elucidate the epigenetic landscape. Additionally, although in vitro models allow precise control of Cd exposure, in vivo studies are required to fully capture the complexity of adipose tissue interactions within the systemic metabolic environment, including we recommend that future studies investigate a diverse donor (including females and individuals with different BMI ranges) to assess potential sex- and metabolic status-dependent effects.

## 4. Materials and Methods

### 4.1. Human Subjects and Sample Collection

Visceral WAT (vWAT) samples were obtained from the omental region using discardable tissue collected during elective surgical procedures. Samples were collected from four male patients aged 30–50 years, with a body mass index (BMI) ≥25 and <27 kg/m^2^, who underwent elective gastric or bariatric surgery at Rede D’Or São Luiz Hospitals (São Paulo, Brazil). All participants provided written informed consent prior to enrollment by signing the Free and Informed Consent Form. The study population was restricted to male patients to minimize potential confounding variables such as sex-related hormonal differences and age-related metabolic variations, which are known to influence adipogenesis and epigenetic regulation. This narrow demographic was intentionally selected to reduce biological variability and establish proof-of-concept in a controlled human primary cell model.

The study protocol was approved by the Research Ethics Committee of the Federal University of São Paulo (CEP/UNIFESP Project No. 0268/2022; approval date: 7 June 2022; CAAE: 57087422.8.0000.5505), and authorization for human tissue collection at Rede D’Or São Luiz Hospitals was granted under Ethics Opinion No. 7.640.160. Participant eligibility was determined according to pre-established inclusion criteria based on World Health Organization (WHO) guidelines.

Immediately after collection, vWAT samples were placed in sterile phosphate-buffered saline (PBS; Sigma-Aldrich, P3813), maintained at 4 °C in an insulated container with ice, to maintain a low temperature (2–8 °C) during transport, a critical step to preserve cell viability by preventing thermal stress and enzymatic degradation, and transported to the Adipose Tissue Physiology and Epigenetics Laboratory at UNIFESP, where they were processed within 2 h of collection.

### 4.2. Adipose Tissue Processing and Cell Isolation

Visceral and subcutaneous white adipose tissue samples were obtained from anatomically defined regions (omental for visceral depots and abdominal for the subcutaneous region) during elective surgical procedures. Tissue processing and cell isolation were performed according to established protocols in the Adipose Tissue Physiology and Epigenetics Laboratory at UNIFESP. Briefly, after dissection and mechanical fragmentation, adipose tissue samples were incubated in digestion buffer composed of Dulbecco’s modified Eagle’s medium (DMEM) supplemented with HEPES (20 mM), bovine serum albumin (BSA, 4%), and collagenase type II (1.0 mg/mL; Sigma Chemical, St. Louis, MO, USA), pH 7.40. Tissue digestion was carried out for approximately 45–60 min at 37 °C in a water bath with orbital shaking (130 rpm). Following digestion, the suspension was filtered through a fine-mesh plastic sieve to remove undigested tissue fragments and vascular debris, and the volume was adjusted to 25 mL with EHB buffer (Earle’s salts supplemented with HEPES 25 mM, BSA 1%, sodium pyruvate 1 mM, glucose-free, pH 7.45, 37 °C). The filtrate was centrifuged at 400× *g* for 1 min, resulting in two distinct fractions: (i) the upper layer containing mature adipocytes, and (ii) the SVF in the infranatant. The SVF is the heterogeneous cellular population obtained after adipose tissue digestion and removal of floating mature adipocytes. The isolation of hASCs from the SVF provides a standard in vitro model to study adipogenesis and cell-specific responses. The SVF was further centrifuged at 1500× *g* for 10 min to obtain a pellet containing stromal cells, including hASCs. The pellet was resuspended and washed twice with EHB buffer using the same centrifugation conditions. Isolated adipocytes and SVF-derived cells were subsequently processed according to the experimental design.

### 4.3. hASCs Culture and Differentiation Protocol

The isolation and expansion of hASCs were performed following our previously described method [19], with specific adaptations. Briefly, the SVF isolated from digested visceral adipose tissue was centrifuged, and the resulting pellet was resuspended in complete culture medium. This medium consisted of DMEM/Ham’s F-12 supplemented with 10% fetal bovine serum (FBS) and 1% penicillin/streptomycin (Gibco, Grand Island, NY, USA). The cell suspension was then plated in culture dishes and maintained at 37 °C in a humidified atmosphere containing 5% CO_2_. The medium was replaced every 48 h. To obtain a purified population of adherent hASCs, the cultures were monitored until they reached 70–80% confluence. At this point, the cells were washed with phosphate-buffered saline (PBS) and detached using trypsin-EDTA, marking the first passage (P1). The cells were then reseeded in fresh complete medium into larger vessels for expansion. Cell counting was performed using a Neubauer chamber. This process of passaging upon reaching 70–80% confluence was repeated consistently, ensuring cells never exceeded 80% confluence to maintain their undifferentiated state. For experimental procedures, cells between passages 2 and 5 (P2–P5) were seeded in 6-well plates (35 mm diameter) at a density of 1 × 10^5^ cells per well. Treatments were administered when the cells reached 85–90% confluence (proliferation assays) or following the completion of differentiation protocols.

To induce adipogenic differentiation, hASCs were first grown to complete (100%) confluence, designated as day 0 (D0). At D0, the standard growth medium was replaced with an adipogenic induction medium. This specialized medium was prepared by supplementing DMEM/Ham’s F-12 with an adipogenic cocktail containing: 0.5 mM 3-isobutyl-1-methylxanthine (IBMX), 0.1 µM dexamethasone, 0.5 µM human insulin, 2 nM triiodothyronine (T3), 30 µM indomethacin, 17 µM pantothenate, 33 µM biotin, 1 µM rosiglitazone, 1 mg/mL apo-transferrin, 2% FBS, and 1% penicillin–streptomycin. The cells were maintained in this induction medium, which was refreshed every 2–3 days, for either 7 or 20 days, as determined by the specific experimental protocol.

### 4.4. Cell Treatments

#### 4.4.1. Cd Treatment

A stock solution of Cd chloride (CdCl_2_, Sigma-Aldrich, St. Louis, MO, USA, purity ≥ 99.9%, Catalog #202908) was prepared at 10 mM in ultrapure water, sterile-filtered (0.22 µm), aliquoted, and stored at −20 °C. For each experiment, fresh working solutions of 1.5 µM and 5 µM CdCl_2_ were prepared in complete culture medium. These concentrations were selected based on preliminary MTT viability assays and existing in vitro literature [13].

The CdCl_2_ treatments were applied according to specific timelines: For undifferentiated, proliferating hASCs, the treatment lasted 10 days. During adipogenic differentiation (induction at Day 0), two protocols were used: (1) Early/Differentiation Phase Treatment: initiated concurrently with the adipogenic cocktail at Day 0 and maintained for 7 days; (2) Late/Post-Differentiation Phase Treatment: initiated on Day 10 post-induction and maintained for 10 days, until the experimental endpoint (Day 20).

In all cases, the culture medium, with or without CdCl_2_, was renewed every 3 days. Specific timelines for each experiment are detailed in the corresponding figure legends.

#### 4.4.2. Treatment with LPS and Combined Treatments

Lipopolysaccharide from *E. coli* (LPS, serotype 055:B5; Sigma, St. Louis, MO, USA) was used at a concentration of 1 μg/mL to stimulate a pro-inflammatory pathway. This concentration was established as a positive control for inflammation in standardization experiments, as it effectively activated the expression of TNF-α and MCP-1 (confirmed by qRT-PCR and ELISA) with minimal impact on cell viability (assessed by MTT assay) [41].

### 4.5. MTT Toxicity Assay

Cell toxicity was evaluated by the 3-[4,5-dimethylthiazol-2-diphenyltetrazolium] formazan bromide reduction method by the MTT cell proliferation Kit (Cat. No. 11465007001, Roche Diagnostics, Mannheim, Germany). Two analyses were performed, the first after 24 h of culture, and the second after 48 h (5 × 10^3^ cells/well in 100 μL D’MEM F-12/FBS) in 96-well plates (flat bottom), 10 μL/well of MTT was added to the cells, which were incubated for 4 h (37 °C, 5% CO_2_). After this period, 100 μL/well of the formazan crystal solubilisation solution (10% SDS in 0.01 M HCl) was added. The plates were then incubated for 12–16 h (37 °C, 5% CO_2_). Results were expressed as percentage values concerning the control.

Four independent human donors (biological replicates) were used for all experiments, with each donor tested in technical triplicate.

### 4.6. RNA Extraction and Real Time PCR

Total RNA was extracted with Trizol reagent (Invitrogen Life Technologies, Waltham, MA, USA), according to the instructions by the provider. Gene expression was evaluated by RT-PCR and the analysis of the results obtained was carried out using the −ΔΔCt method. Data is expressed as the ratio between the expression of the target gene and housekeeping gene (*GAPDH*). The following primers were used: CCL2 (5′-3′ sense: AGAATCACCAGCAGCAAGTGTCC; 5′-3′ antisense: TCCTGAACCCACTTCTGCTTGG); EP300 (5′-3′ sense:TGCAGGCATGGTTCCAGTTT; 5′-3′ antisense:AGGTAGAGGGCCATTAGA AGTCA); EZH2 (5′-3 sense:GCTGGAATCAAAGGATACAGACA; 5′-3′ antisense:GACACCGAGAATTTGCTTCAG); GAPDH (5′-3′ sense:GTCTCCTCTGACTTCAACAGC; 5′-3′ antisense:ACCACCCTGTTGCTGTAGCCAA); KDM6A (5′-3′ sense:GAGGGAAGCTCTCATTGCTG; 5′-3′ antisense:AGATGAGGCGGATGGTAATG); CREBBP (5′-3′ sense:GAAACCAACAAACCATCCTGG; 5′-3′ antisense:CATTGGATTATTTCCCAGGG); CBP (5′-3′ sense:GAAACCAACAAACCATCCTGG; 5′-3′ antisense:CATTGGATTATTTCCCAGGG) ACACA/ACC1 (5′-3′ sense:TGCTGACCGAGAAAGCAGGGGA; 5′-3′ antisense:ACTGGGTCCACCCGACGCAT); ADIPOQ (5′-3′ sense:TCTGCCTTCCGCAGTGTAGG; 5′-3′ antisense: GGTGTGGCTTGGGGATACGA); KLF15 (5′-3′ sense:GGTGAAAAGCGTCCCCCACT; 5′-3′ antisense:TGTCTGGGAAACCGGAGGAG); CEBPA (5′-3′ sense:CAAGAACAGCAACGAGTACCG; 5′-3′ antisense:GTCACTGGTCAGCTCCAGCAC); FABP4 (5′-3′ sense:TCAGTGTGAATGGGGATGTGAT; 5′-3′ antisense:TCTGCACATGTACCAGGACACC); FASN (5′-3′ sense:GATGACCGTCGCTGGAAGG; 5′-3′ antisense:AATCTGGGTTGATGCCTCCG); LEP (5′-3′ sense:ATTTCACACACGCAGTCAGTCT; 5′-3′ antisense:GATAAGGTCAGGATGGGGTGG); PLIN1/Perilipin (5′-3′ sense:GACCTCCCTGAGCAGGAGAAT; 5′-3′ antisense:GTGGGCTTCCTTAGTGCTGG); PPARG (5′-3′ sense:AGAAAGCGATTCCTTCACTGAT; 5′-3′ antisense:AGAATGGCATCTCTGTGTCAAC).

### 4.7. Adipokines Measurements

The concentrations of adipokines from culture supernatant were determined using specific ELISA kits (Quantikine M; R&D Systems, Minneapolis, MN, USA; Catalog numbers DY279-05 (MCP1/CCL2), DY210-05 (TNFA), and DY1065-05 (ADIPOQ), respectively.

### 4.8. Enzyme Activity Assay

Enzymatic assays for CAT (U/mg protein), SOD (Enzymatic activity) and GPx (Enzymatic activity). Antioxidant enzymes were extracted by cell scraping with 150 μL of phosphate buffer (19% KH_2_PO_4_, 81% K_2_HPO_4_ at 7.4 pH). Enzymatic activity was measured utilizing the RANSOD, Cat. No. SD 125/MD kit, for Superoxide Dismutase, RANSEL, Cat. No. RS 504/MD kit (Randox Lab Ltd., County Antrim, UK) for Glutathione Peroxidase, and Catalase activity was measured by comparison with a hydrogen peroxide standard curve and then later normalized by total protein obtained through the bicinchoninic acid (BCA) assay.

### 4.9. Protein Carbonyl Content

Protein carbonyl groups, a marker of protein oxidation, were measured in hASC homogenates using the alkaline 2,4-dinitrophenylhydrazine (DNPH) method [42] (adapted by the Redox-Inflammation Lab, UNESP, São Paulo, Brazil). hASCs were harvested by cell scraping and homogenized in PBS. Aliquots of 100 µL were incubated with 100 µL of 10 mM DNPH prepared in 2 M HCl for 10 min at room temperature in the dark. Subsequently, 50 µL of 6 M NaOH was added, followed by another 10 min incubation in the dark. Absorbance was measured at 450 nm. Carbonyl concentration was calculated using the molar extinction coefficient of 22,000 M^−1^·cm^−1^ and normalized to total protein content, which was determined by BCA assay. Results were expressed as µmol per mg of protein. All experiments were performed with n = 4 from two independent experiments.

### 4.10. Western Blot

Proteins from hASCs treated with different concentrations of Cd were resolved by 12% or 15% sodium dodecyl sulfate (SDS)-polyacrylamide gel electrophoresis (PAGE) and transferred to nitrocellulose membranes (0.2 µm). The membranes were blocked with 5% albumin for 1 h at room temperature and incubated with primary antibodies (anti-H3K27ac #ab4729, H3K27me3 #ab5700166, KDM6A ab#O15550, KDM6B ab#O15054, CREBBP ab#Q92793, EP300 ab#B2RWS6, EZH2 ab #D2C95246S, Ab cam, Waltham, MA, USA) at 4 °C overnight. Membranes were washed and incubated with secondary horseradish peroxidase-conjugated anti-rabbit IgG (Cell Signaling^®^, Danvers, MA, USA—#7074) antibodies at room temperature for 1 h. Protein blots were visualized by using an enhanced chemiluminescence Western Blotting detection kit (ECL Prime Western Blotting System, Amersham Biosciences^®^, Leicestershire, UK). Beta-actin (Cell Signaling^®^, Danvers, MA, USA—#4967L) levels were used as an endogenous standard. Protein quantification was analysed by using Scion Image software version 4.0 (Scion Corporation, Frederick, MD, USA). All the results were expressed relative to control group levels and corrected by the expression of the constitutive beta-actin and total protein by Ponceau.

### 4.11. Statistical Analysis

The data analysis was conducted using a one-way Analysis of Variance (ANOVA), followed by Tukey’s post-test for intergroup comparisons or Student’s *t*-test for comparisons between two groups. The results are presented as the mean ± standard error of the mean (SEM), with significance defined as *p* < 0.05. The statistical evaluations were carried out using GraphPad Prism software, version 9.1.2 (GraphPad Software Inc., San Diego, CA, USA).

## 5. Conclusions

In summary, this study provides the first evidence that non-cytotoxic cadmium exposure modulates H3K27 histone modifiers—particularly KDM6B—in primary human adipose-derived stem cells and differentiated adipocytes. This epigenetic shift occurs alongside redox imbalance (reduced CAT activity, increased protein carbonyls) and a pro-inflammatory response (CCL2 upregulation), ultimately impairing adipogenic differentiation and lipid accumulation. Our findings suggest that even low-level Cd exposure may contribute to adipose tissue dysfunction through interconnected mechanisms involving ROS-driven KDM6B expression and antioxidant disruption. Further studies using genome-wide approaches and in vivo models are needed to fully elucidate the translational implications of these findings.

## Data Availability

The data are available from the corresponding author upon specific request.

## Abbreviations

Cd, cadmium; AT, adipose tissue; hASCs, human adipose stem cells; H3K27, histone H3 lysine 27; mASCs, mesenchymal adipose stem cells; ROS, reactive oxygen species; CAT, catalase; SOD, superoxide dismutase; GPx, glutathione peroxidase; CCL2, C-C motif chemokine ligand 2; EZH2, enhancer of zeste homolog 2; KDM6A, lysine demethylase 6A; KDM6B, lysine demethylase 6B.
